# Long-term efficacy of IL-1 blockers in PAPA patients

**DOI:** 10.1186/1546-0096-13-S1-P207

**Published:** 2015-09-28

**Authors:** M Finetti, R Caorsi, D Marotto, A Buoncompagni, A Omenetti, B Lattanzi, F Minoia, P Picco, M Jorini, A Martini, M Gattorno

**Affiliations:** 1 IRCCS G. Gaslini, U.O. Pediatria II, Genoa, Italy; 2 Asl2 Olbia - distretto Tempio, Ambulatorio Aziendale di Reumatologia, Olbia, Italy; 3 Ospedale Pediatrico G. Salesi, Divisione di Pediatria, Ancona, Italy

## Introduction

PAPA syndrome (Pyogenic Arthritis, Pyoderma gangrenosum, and Acne) is an ultra-rare autosomal dominant, auto-inflammatory disease associated to mutations in the PSTPIP1/CD2BP1 gene. The therapeutic approach during recurrences consists of steroids, while no agreement exists on the chronic management. Evidences on the use of biologics are anecdotal and variable results have been reported.

## Objectives

To evaluate the long-term response to treatment with IL1 antagonist in six patients affected by PAPA syndrome.

## Methods

Six patients (M:F=3:3; 4 pediatric, 1 young adult and 1 adult, mean age 18 years, range 3-50) affected by PAPA syndrome were enrolled and treated with IL1 blockers (5 patients Anakinra, 1 patient Anakinra followed by Canakinumab). Three patients were already treated with anti-TNFα monoclonal antibodies without benefit. Data were collected retrospectively (mean follow-up 26 months, range 4-38). The frequency of articular and cutaneous flares in the 24 months before starting therapy where compared to those occurred during anti-IL1 regimen. Acute phase reactants (ESR, CRP, SAA) were assessed at the last visit before the study enrolment and at last follow-up.

## Results

All the patients displayed a significant decrease in frequency of disease flares (Table 1) and normalization of acute phase reactants. Three patients were asymptomatic during whole follow-up. Patient #5, with a severe and persistent pyoderma gangrenosum, displayed a partial response to Anakinra partially due to a poor compliance to daily s.c. administration. The shift to Canakinumab lead to a fast and complete resolution of the skin manifestations.

**Table 1:** 

Pt	Sex	Mutation	Main manifestations	Manifestations in the 24 months before treatment	N± of flares during follow-up	Treatment (dose, duration)
1	F	E256G	Pyogenic arthritis Pyoderma gangrenosum Cystic acne/foruncolosis	2 flares of pyogenic arthritis 5 pyoderma gangrenosum Cystic acne	0	Anakinra 100 mg/day (36 months)

2	F	E250Q	Pyogenic arthritis Sterile osteomyelitis Palpebral edema	3 flares of pyogenic arthritis 1 sterile osteomyelitis 1 palpebral edema	0	Anakinra 2 mg/kg/day (21 months)

3	M	E250Q	Pyogenic arthritis	7 flares of pyogenic arthritis (polyarticular)	3 (mild) articular flares	Anakinra 1.5 mg/kg/day (38 months) and low dose steroid

4	M	WT	Cutaneous abscesses Pyoderma gangrenosum Pyogenic muscular abscess	1 persistent muscular abscess	0	Anakinra 100 mg/day (26 months)

5	M	E250K	Pyogenic arthritis Pyoderma gangrenosum Severe anemia Splenomegaly Growth delay	1 cutaneous abscess 3 pyoderma gangrenosum Anemia	1 pyoderma gangrenosum (resolved after Canakinumab)	Anakinra 2 mg/kg/day (31 months) - Canakinumab (4 months)

6	F	E250Q	Dactylitis/tendinitis Pyogenic arthritis Acne and furunculosis	6 articular flares	0	Anakinra 100 mg/day (4 months)

## Conclusions

The long-term use of IL1 blockers is associated to satisfactory and persistent control of clinical manifestations and laboratory findings in PAPA syndrome.

